# New for *PCD* in 2022: Increased Impact Factor, Expanded Expertise, First Guest Editorial Board, and Progress in Diversity, Equity, and Inclusion Goals

**DOI:** 10.5888/pcd19.220197

**Published:** 2022-07-28

**Authors:** Leonard Jack

**Affiliations:** 1 Office of Medicine and Science, National Center for Chronic Disease Prevention and Health Promotion, Centers for Disease Control and Prevention, Atlanta, Georgia

This has been an extremely productive year for *Preventing Chronic Disease* (*PCD*). Can I edit this? At the midpoint of 2022 we already have many achievements to recognize and celebrate. Last year we crossed a high bar: in 2021 *PCD* received the biggest jump in impact factor in its history, from 2.144 to 2.803, making it fourth of 27 open-access US public health journals. We are pleased to report that the latest SJR: Scientific Journal Rankings release shows a sustained upward trend, with a new impact factor of 4.354, a 55% increase. Additionally, Scimago Journal Rank recently placed *PCD* fourth out of 30 US journals and 76th out of 585 journals worldwide in the category of Public Health, Environmental, and Occupational Health open access journals. The journal was also ranked in the top 10% of more than 27,000 journals evaluated by Scimago in 2021. 

*PCD* continues its focus on ensuring the highest level of scientific quality and rigor in its content by expanding our expert resources. For our upcoming collection on combating racism, we brought together the journal’s first guest editorial board, whose members bring expertise in areas that intersect issues of racism and public health. We are excited to be working with such a distinguished group. We have also expanded the breadth and depth of representation in our team of associate editors and in our Editorial Board and Statistics Review Committee. We will need the experience and knowledge that all these new experts bring to the journal, in anticipation of another record number of submissions. 

So far this year we have published 2 collections and have two more scheduled. In this column I will share details of these collections and a new collection podcast, as well as our upcoming calls for papers. And finally, I will provide an update on our efforts to advance best practices in diversity, equity, and inclusion (DEI) in our operations and our plans to provide authors with updated guidance on reporting race and ethnicity.

## New Editorial Board, Associate Editor, and Statistics Review Committee Appointees and *PCD*’s First Ever Guest Editorial Board

In 2022 so far, *PCD* has appointed 5 new members to serve on the journal’s Editorial Board. The board is made up of volunteers who advise the journal on its policies and identify appropriate and timely topics for publication. Attention to the latest and most pressing public health topics requires that the journal expand to include the necessary expertise in its Editorial Board. We are pleased to have secured expertise in public health data systems, which will position the journal to disseminate information on how the public health field can use and leverage timely, relevant, and actionable data to generate public health responses (1). *PCD* also identified 3 other areas of growth and has supplemented its expertise to address these important new areas. First, the COVID-19 pandemic has altered the routine of life for many, and those changes have generated unforeseen and severe psychological responses and mental health crises (2). *PCD* has experienced an increase in articles examining various aspects of psychological health and chronic disease and has secured board members with considerable expertise in mental health and psychological well-being. Second, the pandemic has highlighted the importance of technologies, such as telemedicine, and has increased access around the world to mobile and computer devices as a primary source of health information and patient care. To address these topics, *PCD* has secured expertise in health communications, health literacy, and sociotechnical challenges associated with the use of health information technologies. Third, to address the issue of structural determinants of health, *PCD* has appointed new Editorial Board members who work in state and local health departments and have expertise in advancing public health care justice, implementing public health policies, and tackling health care inequities in rural and urban settings. Please learn more about members serving on the journal’s Editorial Board by visiting https://www.cdc.gov/pcd/about_the_journal/editorial_board.htm.

In addition to appointing new experts to the Editorial Board, we have also appointed several new associate editors. The job of associate editors is to determine whether manuscripts submitted to the journal are appropriate to undergo peer review. If so, associate editors facilitate peer review and recommend acceptance to the editor in chief. This year’s appointees expand the journal’s expertise in areas such as the built environment’s effect on COVID-19 and chronic disease and the application of informatics in surveillance, prevention, preparedness, and health promotion. Also, over the past 2 years, *PCD* has received an increased number of articles reporting on disease management programs among military services members and their families, requiring expertise in these public health issues. New information continues to emerge on how and to what extent the COVID-19 pandemic has exacerbated existing health inequities among certain racial and ethnic groups, including African Americans, Hispanic, and American Indian people, especially those living with chronic disease, which requires expertise in these areas. We have acquired associate editors with knowledge and experience in contact tracing, viral infection, respiratory illness, COVID-19 infections in health care settings, and behavioral and social drivers of COVID-19 vaccination. To learn more about the journal’s associate editors, please visit https://www.cdc.gov/pcd/about_the_journal/associate_editors.htm.

And finally, *PCD* created its first guest editorial board for an upcoming collection on combating racism that addresses the impact of all forms of racism on health. The journal brought together a group of distinguished individuals from across the US with experience and expertise in addressing racism in relevant areas such as epidemiology, clinical practice, health communications, community and health department collaborations, and public health policy. We will look to this guest editorial board to assist the journal’s efforts in providing guidance to authors on how best to report on race and ethnicity in articles submitted to the journal for consideration. You can learn more about the guest editorial board and read the call for papers for the forthcoming collection, *Public Health, Medicine, Dentistry, Nursing, and Pharmacy: Combating Racism Through Research, Training, Practice, and Public Health Policies*, by visiting *PCD*’s Announcements page: https://www.cdc.gov/pcd/announcements.htm.

*PCD*’s Statistics Review Committee, our most recent creation, was formed in 2019 to address the increasing complexity of statistical methods in articles submitted to us. Committee members are volunteers with training and expertise in statistics and biostatistics who assist the journal in peer review to determine the appropriateness of research and evaluation questions, selection of study sample, statistical tests used, and analysis of data based on research design, distribution of data, and type of variables under examination. *PCD* appointed 5 new members this year to the Statistics Review Committee with expertise in biostatistics and informatics, conceptualizing and conducting research on race and ethnicity, multilevel modeling and techniques for dimension reduction and modeling time-intensive longitudinal data, Bayesian data analysis, clinical trial studies, and genomics. You can learn more about members of the journal’s Statistics Review Committee by visiting https://www.cdc.gov/pcd/about_the_journal/Statistics_Review_Committee.htm.

## *PCD* Collections and Calls for Papers

### Collections for 2022

*PCD* remains committed to publishing collections, or supplements, that group together published articles under specific topics and themes for rapid dissemination to our readers. In April 2022, we published a collection entitled *Cancer Screening Prevalence and Associated Factors Among US Adults*. Cancer is the second leading cause of death in the United States, exceeded only by heart disease (2). Understanding cancer screening patterns and factors associated with getting screened helps public health policy makers and practitioners improve cancer prevention programs by implementing evidence-based policies and practices. This collection features 11 research articles on cancer screening trends, determinants of cancer screening, and public health practices that increase cancer screening uptake in specific populations (3). Guest editors for this collection are Zhen-Qiang (Marshal) Ma, MD, MPH, MS, director of the Division Of Community Epidemiology at the Pennsylvania Department of Health, and Lisa C. Richardson, MD, MPH, director of the Division of Cancer Prevention and Control at the Centers for Disease Control and Prevention. We are particularly excited to include with this collection *PCD*’s first podcast of 2022, a discussion with Dr Richardson and Dr Ma about the collection’s relevance to issues surrounding cancer screening and prevention from a public health perspective.

*PCD* recently released its second collection of 2022, *Global Perspectives on the Intersection of Chronic Disease and COVID-19*, featuring 11 articles that highlight the various ways geographic information systems, spatial analysis, and other geospatial techniques and technologies are applied to research and public health practice (4). We assembled an excellent group of guest editors for this collection: Jeremy Mennis, PhD, Professor, Geography and Urban Studies Department, Temple University; Sarah Huston, PhD, Lead Chronic Disease Epidemiologist, Maine Center for Disease Control and Prevention; and Kevin Matthews, PhD, MS, Health Geographer, National Center for Chronic Disease Prevention and Health Promotion, Centers for Disease Control and Prevention.

*PCD* continues its commitment to student publishing by offering high school, undergraduate, graduate, doctoral, and postdoctoral students the opportunity to submit manuscripts to the journal for consideration. In September we will release a collection of articles by students who serve as first and corresponding authors of original research. Our student articles released to date address topics relevant to the prevention, screening, and surveillance of population-based interventions for chronic diseases and cover topics such as arthritis, cancer, diabetes, depression, obesity, and cardiovascular disease.

Our final collection of the year addresses global responses to prevent, manage, and control cardiovascular disease. Look for that collection in December, with guest editors Fatima Coronado, MD, MPH, Associate Director for Science in CDC’s Division for Heart Disease and Stroke Prevention; Guixiang (Grace) Zhao, PhD, Health Scientist in CDC’s Division of Population Health; and Mario Sims, PhD, MS, FAHA, Professor of Medicine in the Department of Medicine at the University of Mississippi Medical Center and Chief Science Officer of the Jackson Heart Study.

### Calls for papers for 2022

*PCD* continues to identify emerging areas of research, evaluation, and public health practice of interest to the journal’s readership. We currently have 7 active calls for papers (see Announcements at https://www.cdc.gov/pcd/announcements.htm) and encourage interested authors to submit manuscripts for consideration in the following areas:

- **Global Responses to Prevent, Manage, and Control Cardiovascular Disease** (https://www.cdc.gov/pcd/announcements.htm#global_response). Heart disease and stroke continue to be leading causes of death and disability worldwide, exceeding even the recent impact of the COVID-19 pandemic (5). *PCD* is interested in publishing peer-reviewed articles from around the world offering insight into successes and challenges of public health strategies implemented to improve cardiovascular health through prevention, detection, and treatment.
- **Health Equity in Action: Research, Policy, and Practice** (https://www.cdc.gov/pcd/announcements.htm#health_equity). According to Healthy People 2020, achieving health equity requires valuing everyone equally with focused and ongoing societal efforts to address avoidable inequities, historic and contemporary injustices, and the elimination of health and health care disparities (6). *PCD* is soliciting manuscripts that explore implementation of fair, restorative, and equitable interventions to address long-standing chronic disease prevalence among socioeconomically disadvantaged populations.
- **Public Health, Medicine, Dentistry, Nursing, and Pharmacy: Combating Racism Through Research, Training, Practice, and Public Health Policies** (https://www.cdc.gov/pcd/announcements.htm#combatRac). In April 2021, CDC declared racism a public health threat, identifying it as one of the fundamental drivers of health inequities (7). *PCD* is interested in publishing peer-reviewed articles offering insightful methodologic approaches to measuring racism across the lifespan, the impact of racism on physical and mental health, interventions that affect health care providers and can lead to improvement in patient outcomes, and differential effects of institutional racism on policies, practices, and laws and how they affect members of certain racial groups.
- **Sleep Deprivation, Sleep Disorders, and Chronic Disease** (https://www.cdc.gov/pcd/announcements.htm#sleepDep). Sleep is an essential daily behavior that supports physical, emotional, and psychological well-being (8). Nearly every system of the body depends on satisfactory sleep quality and quantity for routine healing, repair, and restoration (8). *PCD* seeks to publish peer-reviewed articles that increase awareness among clinicians, public health experts, and patients regarding the relationship between sleep and chronic disease. This collection will also offer insights into successes and challenges of public health strategies to improve quality of sleep.
- **Implementing and Sustaining Policy, Systems, and Environmental Changes to Support Healthy Behaviors in Diverse Settings** (https://www.cdc.gov/pcd/announcements.htm#capImpSus). Health behaviors are shaped not only by individual choices but also by the environments in which they occur (9). Policy, system, and environmental changes seek to create conditions that support people in adopting and sustaining healthy behaviors (10). *PCD* is interested in articles that describe the use and effectiveness of such changes in creating accessible and available settings, environments, and conditions that help people make healthy choices in schools, communities, worksites, hospitals, restaurants, and beyond.
- **Tools and Techniques to Conduct Tailored Performance Monitoring and Evaluation** (https://www.cdc.gov/pcd/announcements.htm#capTools). Assessing the impact of public health interventions is important for several reasons. Typically, fiscal resources are used to support public health interventions, so it is imperative to determine whether the return on those investments supports changes in how these interventions are delivered or whether the interventions should be discontinued altogether (11). This collection will feature peer-reviewed articles in the journal’s category, Tools for Public Health Practice. This article type provides instructional content to support professional development and focuses on the “how-to,” practical application of public health methods.
- **Advancing Chronic Disease Data Modernization Enhancements to Meet Current and Future Public Health Challenges** (https://www.cdc.gov/pcd/announcements.htm#capAdvMod). The nation’s health data systems are often antiquated, resulting in a myriad of negative effects on chronic disease prevention and health promotion efforts. In addition, antiquated data management systems often become difficult to support, maintain, scale, or integrate into new platforms (12). *PCD* is interested in publishing articles describing and highlighting data-management infrastructure transformations that support response-ready systems to meet not only today’s health challenges but tomorrow’s as well. This collection will feature peer-reviewed articles showcasing efforts underway by federal, state, tribal, local, and territorial public health agencies to collect, use, and share data.

## Progress on Diversity, Equity, and Inclusion Efforts

As outlined in our Position Statement, *PCD* is in the process of implementing its strategic plan to advance DEI by continued expansion in 4 key areas (13):

- Scientific leadership
- The peer-review process, including enlarging the pool of authors and peer reviewers
- Expanding research to identify potentially effective ways to improve health equity and shed light on the intersection of racism and health
- DEI-related training and continuing education opportunities among *PCD* staff members, volunteers, peer reviewers, and authors

We have already made progress in all of these areas. The journal has expanded its scientific leadership by increasing and broadening its expert boards and committees that support the journal’s efforts to shed light on the intersection of racism and health. In this year’s calls for papers, *PCD* seeks an in-depth exploration of ways to combat racism, advance health equity, and address the roots of health disparities with honesty and courage. In addition to adding these knowledgeable and dedicated experts to the *PCD* team, our internal staff of *PCD* editors has implemented the latest inclusive language guidance from the American Medical Association and from CDC’s Health Equity Office and regularly pursues training on DEI-related topics in scientific editing and publication to ensure that we publish content that is sensitive and inclusive.

Finally, over the past year, I have had the pleasure of serving as co-chair of the Council of Science Editor’s (CSE’s) Diversity, Equity, and Inclusion Committee. The purpose of this committee is to ensure that CSE reflects the best, most earned, most equitable, and fair representation of both the profession and the work published in scholarly communications (14). CSE is committed to providing its members with DEI-related training and resources. It has been a pleasure leading some of these efforts as co-chair of CSE’s DEI Committee. One such example involved securing and leading a panel of four journal editors-in-chief who spoke at this year’s CSE annual meeting on establishing and sustaining the expansion of diversity, equitable decision making, and a culture of inclusion in scholarly communications and between journal publishing professionals. Editors in chief from the following journals participated on the panel: Preventing Chronic Disease: Public Health Research, Practice and Policy; American Family Physician; Health Education & Behavior; and the American Journal of Public Health. Editors in chief identified several strategies for advancing DEI principles in scholarly publications that include encouraging expansion of content about the importance of addressing racism and social determinants of health, hiring DEI associate editors, ensuring diversity among volunteers (editorial boards, associate editors, and peer reviewers), revising journal editorial policies to integrate DEI best practices, acknowledging delays and missteps to help ensure transparency and public trust, and the need for journals to remain in close communication and to share lessons learned with one another (15). *PCD* will continue to help lead national discussions and action steps to advance DEI approaches in scholarly publishing. We look forward to keeping our readership up to date on these efforts moving forward.

Over the coming months, we expect to begin collecting demographic data from users of *PCD*’s ScholarOne manuscript submission system. Users of the system will be prompted upon login to address incomplete profile information, including providing additional demographic details. To protect users’ privacy, responses are voluntary and will be viewable only in the aggregate by *PCD* and ScholarOne staff. Initial questions will address gender identity, race, and ethnicity to help us better understand the diverse representation of our contributors and reviewers and guide our future efforts. We hope to include additional questions in future phases. *PCD* readers will also receive updates on the journal’s guidance to authors regarding the reporting of race and ethnicity in our articles. Specifically, the journal seeks to provide the best guidance on how the Methods section of manuscripts submitted to *PCD* in original research articles and briefs should explain who identified the participant race or ethnicity and the source of the classifications used. In addition, *PCD* will require authors to provide a rationale as to why race and ethnicity categories were collected for a study and to report the race and ethnicity of study populations in the Results section.

## Conclusion

At this midpoint of the year, *PCD* already has an impressive list of accomplishments, thanks to the combined expertise and commitment of our volunteers, authors, and peer reviewers and our excellent staff. As pleased as we are with our progress, we are even more excited about the plans ahead. Until the next update, we ask for your continued support in sharing with your colleagues the many calls for papers presented in this column. If you find one or more of the topics interesting, please think about submitting your work to *PCD* for consideration. As always, we thank the journal’s increasing number of volunteers who serve as associate editors, Editorial Board members, Statistics Review Committee members, and peer reviewers for positioning the journal to experience continued success.

## References

[1] Evans AC , Bufka LF . The critical need for a population health approach: addressing the nation’s behavioral health during the COVID-19 pandemic and beyond. Prev Chronic Dis 2020;17:E79. 10.5888/pcd17.200261 32762806 PMC7417017

[2] US Cancer Statistics Working Group. US cancer statistics: data visualizations. Cancer statistics at a glance. US Department of Health and Human Services, Centers for Disease Control and Prevention, and National Cancer Institute. Released in June 2021. www.cdc.gov/cancer/dataviz. Accessed June 15, 2022.

[3] Ma ZQ , Richardson LC . Cancer screening prevalence and associated factors among US adults. Prev Chronic Dis 2022;19:E22. 10.5888/pcd19.220063 35446757 PMC9044902

[4] Mennis J , Matthews KA , Huston SL . Geospatial perspectives on the intersection of chronic disease and COVID-19. Prev Chronic Dis 2022;19:E39. 10.5888/pcd19.220145 35772034 PMC9258441

[5] Virani SS , Alonso A , Aparicio HJ , Benjamin EJ , Bittencourt MS , Callaway CW , ; American Heart Association Council on Epidemiology and Prevention Statistics Committee and Stroke Statistics Subcommittee. Heart disease and stroke statistics — 2021 update: a report from the American Heart Association. Circulation 2021;143(8):e254–743. 10.1161/CIR.0000000000000950 33501848 PMC13036842

[6] Healthy People 2020. Disparities. Washington (DC): US Department of Health and Human Services, Office of Disease Prevention and Health Promotion. https://www.healthypeople.gov/2020/about/foundation-health-measures/Disparities#:~:text=Healthy%20People%202020%20defines%20health%20equityas%20the%20%E2%80%9Cattainment,the%20elimination%20of%20health%20and%20health%20care%20disparities.%E2%80%9D5. Accessed June 15, 2022.

[7] Walensky RP . Racism is a serious threat to the public’s health. https://www.cdc.gov/healthequity/racism-disparities/index.html. Accessed June 15, 2022.

[8] Watson NF , Badr MS , Belenky G , Bliwise DL , Buxton OM , Buysse D , ; Consensus Conference Panel; Non-Participating Observers; American Academy of Sleep Medicine Staff. Recommended amount of sleep for a healthy adult: a joint Consensus Statement of the American Academy of Sleep Medicine and Sleep Research Society. J Clin Sleep Med 2015;11(6):591–2. 10.5664/jcsm.4758 25979105 PMC4442216

[9] Lyn R , Aytur S , Davis TA , Eyler AA , Evenson KR , Chriqui JF , Policy, systems, and environmental approaches for obesity prevention: a framework to inform local and state action. J Public Health Manag Pract 2013;19(3 Suppl 1):S23–33. 10.1097/PHH.0b013e3182841709 23529052 PMC4943076

[10] Burke MP , Gleason S , Singh A , Wilkin MK . Policy, systems, and environmental change strategies in the Supplemental Nutrition Assistance Program-Education (SNAP-Ed). J Nutr Educ Behav 2022;54(4):320–6. 10.1016/j.jneb.2021.09.008 35027308

[11] Kidder DP , Chapel TJ . CDC’s program evaluation journey: 1999 to present. Public Health Rep 2018;133(4):356–9. 10.1177/0033354918778034 29928844 PMC6055298

[12] Centers for Disease Control and Prevention. Data modernization in action. https://www.cdc.gov/csels/dmi-support/data-modernization-in-action/index.html#:~:text=Data%20Modernization%20in%20Action%20CDC%20and%20partners%20are,prevent%20disease%20from%20occurring%20in%20the%20first%20place. Accessed June 15, 2022.

[13] Jack L Jr . PCD’s Commitment to advancing diversity, equity, and inclusion in its scientific leadership, peer-review process, research focus, training, and continuing education. Prev Chronic Dis 2021;18:E80. 10.5888/pcd18.210269 34387186 PMC8388199

[14] Schmidt MS , Iwuchukwu O . Iwuchukwu. Diversity, Equity, and Inclusion (DEI) Task Force update and future direction. Sci Ed 2021;44(3):84–5. 10.36591/SE-D-4403-84

[15] King A . Approaches to advancing diversity, equity, and inclusion in journal publishing. Science Editor. https://www.csescienceeditor.org/article/approaches-to-advancing-diversity-equity-and-inclusion-in-journal-publishing/. Accessed July 18 2022.

